# A novel accessory protein ArCel5 from cellulose-gelatinizing fungus *Arthrobotrys* sp. CX1

**DOI:** 10.1186/s40643-022-00519-1

**Published:** 2022-03-21

**Authors:** Yue Yuan, Chunshu Chen, Xueyan Wang, Shaonian Shen, Xiaoyu Guo, Xiaoyi Chen, Fan Yang, Xianzhen Li

**Affiliations:** School of Biological Engineering, Dalian Polytechnic University, Ganjingziqu, Dalian 116034 People’s Republic of China

**Keywords:** Accessory protein, CBM, Linker, Glycosylation, Decrystallization, Synergism

## Abstract

Improved understanding of cellulose swelling mechanism is beneficial for increasing the hydrolysis efficiency of cellulosic substrates. Here, we report a family 5 glycoside hydrolase ArCel5 isolated from the cellulose-gelatinizing fungus *Arthrobotrys* sp. CX1. ArCel5 exhibited low specific hydrolysis activity and high cellulose swelling capability, which suggested that this protein might function as an accessory protein. Homology modeling glycosylation detection revealed that ArCel5 is a multi-domain protein including a family 1 carbohydrate-binding module, a glycosylation linker, and a catalytic domain. The adsorption capacity, structural changes and hydrature index of filter paper treated by different ArCel5 mutants demonstrated that CBM1 and linker played an essential role in recognizing, binding and decrystallizing cellulosic substrates, which further encouraged the synergistic action between ArCel5 and cellulases. Notably, glycosylation modification further strengthened the function of the linker region. Overall, our study provides insight into the cellulose decrystallization mechanism by a novel accessory protein ArCel5 that will benefit future applications.

**Graphical Abstract:**

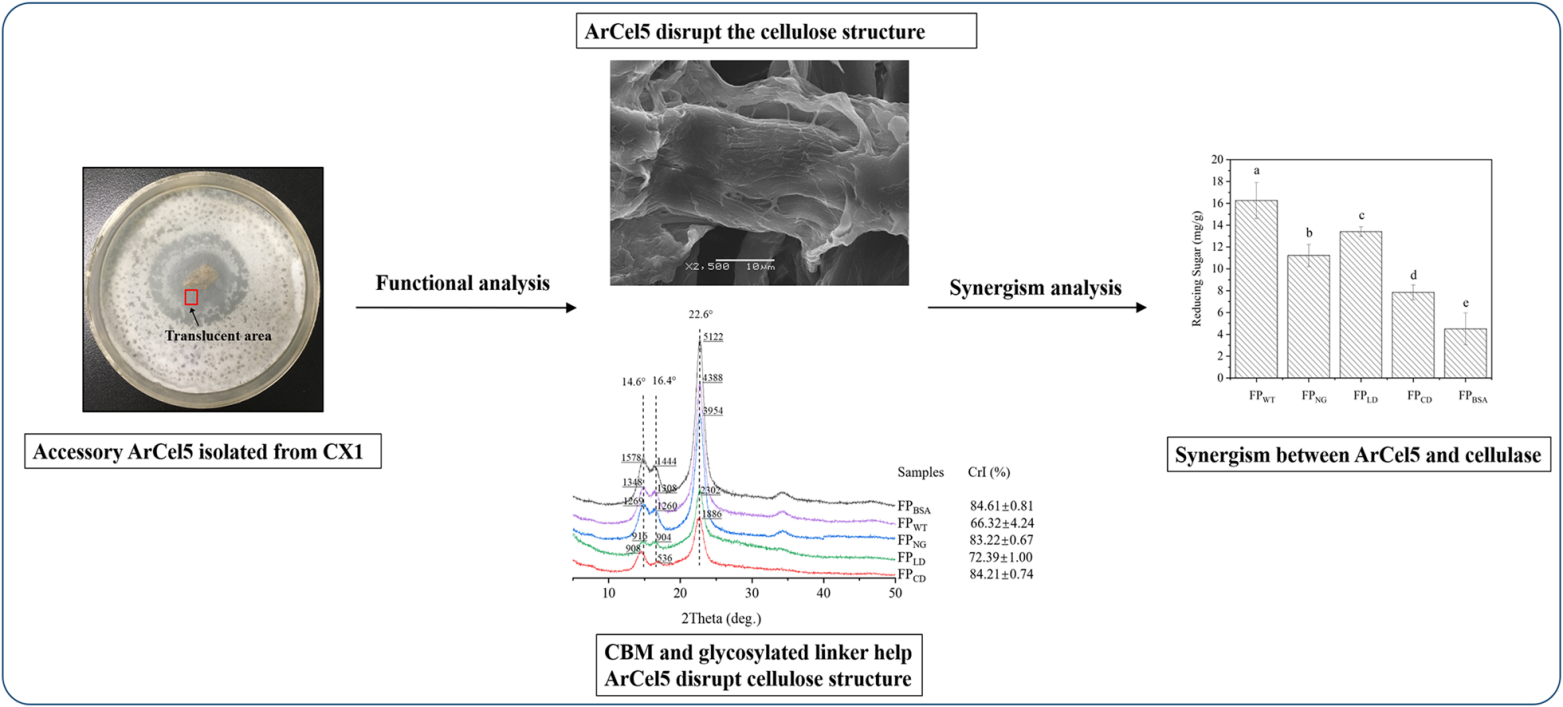

**Supplementary Information:**

The online version contains supplementary material available at 10.1186/s40643-022-00519-1.

## Introduction

The accessibility of cellulose limits the enzymatic saccharification efficiency of cellulose (Arantes and Saddler 2010). The high crystallinity of cellulose could lead to the decrease of enzymatic accessibility, which subsequently becomes the bottleneck for effective hydrolysis of cellulosic substrates (Rollin et al. 2011; Mansfield et al. 2010). Besides engineering cellulases with higher activities (Chundawat et al. 2011; Prajapati et al. 2018), decreasing the crystallinity and increasing the accessibility of cellulose is essential for improving enzymolysis efficiency (Eibinger et al. 2016; Hu et al. 2013; Qin et al. 2013; Song et al. 2018). For this reason, the searching of accessory proteins with effective decrystallization ability is valuable. Mandel and Reese (Reese et al. 1950) previously proposed that a non-hydrolytic swelling factor C_1_ destroys the crystal structure of cellulose, allowing accessible cellulose to be depolymerized by the hydrolytic enzymes (*C*_x_). At present, a series of *C*_1_ like accessory proteins, such as LPMO, swollenins and expansins, have been proved to loosen and deconstruct the crystalline network of cellulose, facilitating the hydrolysis action of cellulases (Arantes and Saddler 2010; Horn et al. 2012; Payne et al. 2015). However, the main factor influencing the function of accessory protein has not been clearly explained (Arantes and Saddler 2010; Eibinger et al. 2016; Saloheimo et al. 2002).

Many accessory proteins exhibit multi-modularization structure, including a carbohydrate-binding module (CBM) and a catalytic domain connected by a flexible linker (Eibinger et al. 2016; Nikolaos et al. 2015; Saloheimo et al. 2002). CBMs are widely distributed in proteins related to cellulose degradation, and play an essential role in adsorption on insoluble substrates (Gilbert et al. 2013). It is known that CBM and catalytic domain could bind to cellulose, but the affinity of CBM is much higher than that of the catalytic domain (Ståhlberg et al. 1991). Generally, the linker is considered as a disordered polypeptide that maintains the flexibility of protein and connects domains with specific functions (Beckham et al. 2010; Receveur et al. 2002). Usually, the linker is proved to contain more glycosylation sites (Payne et al. 2015; Sammond et al. 2012), which could improve the solubility and stability of the protein (Amore et al. 2017; Gupta et al. 2011). Additionally, recent studies demonstrated that O-glycosylated linker also plays a particular role in the binding process of cellulase and substrate (Amore et al. 2017; Badino et al. 2017; Payne et al. 2013). However, the fundamental effect of the multi-domain structure and post-translational modification on accessory proteins remains unclear.

This study isolated a novel accessory protein ArCel5 from a cellulose-gelatinizing fungus *Arthrobotrys* sp. CX1. To explain the critical factor that affects the decrystallization ability of accessory proteins, the structure, glycosylation sites, and the cellulose-decrystallizing properties of the ArCel5 were analyzed, the effect of CBM and glycosylated linker on the properties of ArCel5 are also investigated and discussed. Our findings should shed light on the molecular mechanism of the cellulose decrystallization process.

## Materials and methods

### Plasmids, strains and culture conditions

*Escherichia coli* DH5α was used in all cloning experiments. The plasmid pPICZαA and wild-type *Pichia pastoris* X-33 were used for the eukaryotic expression of ArCel5 and mutants, whereas the plasmid pET28a and *Escherichia coli* BL21 (DE3) were ready for the prokaryotic expression of ArCel5. All the above plasmids and strains were kind gifts from Prof. Zhao (Dalian Institute of Chemical Physics, CAS). Cellulose-gelatinizing strain *Arthrobotrys* sp. CX1 was previously isolated by the author’s laboratory (Lan et al. 2016) and used in this study for cloning of ArCel5-encoding genes.

*Arthrobotrys* sp. CX1 was cultivated at 30 ℃ for 8–10 days on the filter paper agar plate (per liter was composed of 2 g yeast extract and 20 g agar in the mineral salt solution to produce mineral salt agar plate, and a piece of Whatman filter paper on the mineral salt agar plate). The mineral salt solution (per liter was composed of 2 g MgSO_4_·7H_2_O, 2 g Na_2_HPO_4_, 0.2 g FeSO_4_·7H_2_O, 0.7 g CaCl_2_, at pH 6.8–7.0) supplemented with 100 mg of ampicillin and 30 mg of chloramphenicol. Until the translucent paper was observed, a small piece of translucent filter paper (0.5 cm × 0.5 cm) from the colony edge was transferred into 100 mL microcrystalline cellulose medium (per liter was composed of 2 g yeast extract and 10 g Avicel PH101 microcrystalline cellulose in the mineral salt solution). Then *Arthrobotrys* sp. CX1 was cultivated at 30℃ for 7 days in 250-mL flasks on a rotary shaker incubated at 200 rpm. *E. coli* strains were grown at 37 ℃ in LB medium (per liter was composed of 10 g tryptone, 5.0 g yeast extract, 10 g sodium chloride at pH 7.0) supplemented with 25 μg/mL zeocin or 100 μg/mL kanamycin if necessary. *Pichia pastoris* strains were cultured at 30 ℃ in YPD medium (per liter was composed of 10 g yeast extract, 20 g peptone, 20 g dextrose, at pH 6.0), BMGY medium (per liter was composed of 3.4 g YNB, 10 g ammonium sulfate, 10 g glycerol, 10 g yeast extract, 10 g peptone, 100 mL of 1 M phosphate buffer, pH 6.0, and 2 mL of 200 g/L D-biotin), and BMMY medium (identical to BMGY except that it contains 10 mL/L of methanol instead of glycerol).

### Analysis of the composition of potential accessory protein by MS-based

*Arthrobotrys* sp. CX1 was cultured on a filter paper agar plate for 10 days at 30 ℃, and the filter paper samples in the degraded area (transparent) and the undegraded area (opaque) were collected and analyzed by SDS-PAGE. The Micro Protein PAGE Recovery Kit (Sangon Biotech, China) was used to recover differentially expressed proteins. The differentially expressed protein was dissolved in buffer I (8 M urea + 50 mM Tris–HCl, pH 8.0). After that, the proteins were reduced by 20 mM DTT at 60 ℃ for 1 h, after cool down to room temperature, alkylated by 20 mM IAA at room temperature for 40 min in the dark, then diluted to buffer II (1 M urea + 50 mM Tris–HCl, pH 8.0). Trypsin (protease concentration 1 mg/mL) was added with an enzyme to protein mass ratio of 1:20, and the peptides were obtained by incubation at 37 ℃ for 20 h. The tryptic peptides were desalted by the Oasis HLB column (Waters, America) and dissolved in 0.1% FA after vacuum concentration. Peptides were loaded onto a reversed-phase pre-column (Acclaim PepMap 100, Thermo Scientific), then peptides were separated by an Acclaim PepMapTM RSLC reversed-phase analytical column. The analysis of peptides by using Q-Exactive mass spectrometer equipped with a nanospray ion source and an Ultimate 3000 RSLC nano System. Using MS/MS data obtained by Proteome Discoverer 2.2.0.388. MS identification was performed as described previously (Sun et al. 2019).

### Protein sequence analysis and molecular modeling

Sequence alignment was analyzed by Clustal Omega (Sievers et al. 2011), and the sequence alignment results were output by Espript 3 server (Patrice et al. 2003). The structure of ArCel5 was predicted by using the I-TASSER protein structure homology-modeling online server (http://zhanglab.ccmb.med.umich.edu/I-TASSER/). Models were visualized with PyMOL (http://pymol.sourceforge.net/). The molecular weight of the protein is estimated on the tool page of ExPASy (http://www.expasy.org/tools/). The signal peptide of the gene encoding wild-type ArCel5 was predicted by SignalP 4.0 (Petersen et al. 2011). NetOGlyc (http://www.cbs.dtu.dk/services/NetOGlyc/) and NetNGlyc (http://www.cbs.dtu.dk/services/NetNGlyc/) servers were used to predict glycosylation sites.

### Expression and purification of ArCel5 and its mutants

After 7 days of cultivation in microcrystalline cellulose medium, the cells of *Arthrobotrys* sp. CX1 were collected and washed repeatedly with double distilled water. Total RNA of *Arthrobotrys* sp. CX1 was isolated using RNAiso Plus (TaKaRa Bio Inc., Dalian, China) according to the manufacturer’s protocol. The RNA was reverse-transcribed into cDNA using a PrimeScript™ One Step RT-PCR Kit Ver.2 (TaKaRa Bio Inc., Dalian, China) according to the manufacturer’s instructions, and the recombinant plasmid was amplified by PCR using PrimeSTAR HS DNA Polymerase (TaKaRa Bio Inc., Dalian, China) and the following primers (Additional file 1: Table S1). The native signal peptide of the ArCel5 was removed from all recombinants. These PCR products of different lengths gene encoding ArCel5 were digested with *Eco*RI and *Xba*I and ligated into *Eco*RI-*Xba*I-cut pPICZαA to construct recombinant plasmids pPICZαA-ArCel5, pPICZαA-ArCel5-NG, pPICZαA-ArCel5-LD, pPICZαA-ArCel5-D. The PCR product of *arcel5* was digested with *Eco*RI and *Hin*dIII and ligated into *Eco*RI-*Hin*dIII-cut pET28a to construct recombinant plasmid pET28a-ArCel5. pPICZαA-ArCel5, pPICZαA-ArCel5-NG, pPICZαA-ArCel5-LD, pPICZαA-ArCel5-D after digested by *Sac*I, linearized recombinant plasmids were transformed into *P. pastoris* X-33 to express ArCel5 mutants, respectively. As shown in Fig. 5a, pPICZαA-ArCel5 was used to express ArCel5 (Pp-ArCel5), pPICZαA-ArCel5-NG was used to express the nonglycosylated mutant (Pp-ArCel5-NG) that has a linker with mutation all of the Thr residues to Ala, pPICZαA-ArCel5-LD was used to express a mutant with the removal of CBM1 (Pp-ArCel5-LD), pPICZαA-ArCel5-D was used to express a mutant with the removal of CBM1 and linker (Pp-ArCel5-D), the above four plasmids after digested by *Sac*I, linearized recombinant plasmids were transformed into *P. pastoris* X-33 to express ArCel5 mutants, respectively. pET28a-ArCel5 was transformed into *E. coli* BL21 (DE3) used to express ArCel5 (Ec-ArCel5). Transformation methods referred to the Invitrogen *Pichia pastoris* expression manual and pET system operation manual.

For the expression of ArCel5 and its mutants in *P. pastoris* X-33, the recombinant cells were first grown in 5 mL YPD medium containing zeocin (100 mg/mL), then incubated at 30 ℃ and 200 rpm. After 12 h, 0.5 mL cultures were inoculated to 50 mL BMGY medium in 500 mL shake flasks at 30 ℃ and 200 rpm for 16–18 h until OD_600_ reached 4.0. Cells were collected by centrifugation at room temperature, resuspended in 200 mL of BMMY medium in a 2-L baffled flask, and then incubated at 30 ℃ for 96 h. Methanol was added every 24 h with the final concentration of 1% (v/v) to maintain induction of ArCel5. The culture supernatants were collected by centrifugation at 8000 g for 30 min at 4℃. The fermentation supernatant was filtered through a 0.22 μm polyethersulfone membrane. The concentrated enzyme solutions were purified by a 10-kDa molecular weight cutoff membrane (Millipore Corporation, USA).

For the expression of ArCel5 in *E. coli* BL21 (DE3), transformants were grown at 37 ℃ in 200 mL LB medium supplemented with 50 mg/L kanamycin until OD_600_ reached 0.5 and induced by adding IPTG at a final concentration of 0.5 mM, after which the cells were further cultured at 16 ℃ for 14 h. Cells were harvested by centrifugation, and the cell pellet was suspended and sonicated 15 min on ice for 1 s with 3 s intervals in between with an ultrasonic processor. Following the removal of cell debris by centrifugation, the supernatant was ready for subsequent purification. Subsequently, His-tagged proteins were purified by the Ni–NTA purification system using an AKTA Prime Plus (GE Healthcare) as described previously (Crowe 1994). 

### Comparative analysis by SDS-PAGE

According to the method described by Laemmli (Laemmli 1970), protein samples were mixed with 5 × loading buffer and incubated at 95 ℃ for 10 min prior to being analyzed on 12% (w/v) polyacrylamide gel. The gel was stained with Coomassie brilliant blue R-250, and the stained protein gel was decolorized with decolorizing solution (5% ethanol, 10% acetic acid). Glycosylation staining used the Pierce™ Glycoprotein Staining Kit (Thermo Fisher Scientific), following the manufacturer’s instructions. Western blot was analyzed as previously reported (Effenberger et al. 2017).

### Characterization of binding affinity

The adsorption experiment was carried out in a 2-mL tube. Incubate the accessory protein with 10 mg/mL Avicel and filter paper substrates, which were dissolved in 50 mM acetic acid–sodium acetate buffer (pH 5.0). The protein concentration was 0.1–1 mg/mL, the total volume was 0.5 mL, the adsorption temperature was 25 °C, and the substrate was shaken at 120 rpm for 1 h to ensure the adequate combination of protein and substrate. Centrifuge (5 min, 12,000*g*, room temperature) and measure the content of unbound protein with Bio-Rad Protein Assay Kit (Bio-Rad, USA). The Langmuir adsorption isotherms of filter paper and Avicel were selected by appropriate fit (*R*^2^ ≥ 0.98). To study the role of different domains in binding to cellulose substrate, Pp-ArCel5, Pp-ArCel5-NG, Pp-ArCel5-LD, Pp-ArCel5-D, and cellulose samples were incubated under the same conditions. The adsorbed accessory protein concentration was calculated as the difference between the total protein and the unbound accessory protein concentration. The adsorption isotherm parameters were determined by Langmuir isotherm (Arola and Linder 2016; Eibinger et al. 2016), where *B* represents the adsorption amount of protein per gram of cellulose (μmol/g), *B*_max_ represents the maximum adsorption amount of protein per gram of cellulose in an equilibrium state (μmol/g), and *K*_d_ represents dissociation constant (μmol/L).

### Scanning electron microscopy analysis

Scanning electron microscopy (SEM) (JEOL, Japan) was applied to the microstructural changes and surface characteristics of Pp-ArCel5 treatment on filter paper. The filter paper used as control was treated with BSA in 50 mM acetic acid–sodium acetate buffer (pH 5.0). Before SEM evaluation, the dried samples were coated with a thin layer of gold to prevent the sample from becoming charged under the electron beam.

### Enzyme assay

The specific activity of the enzymes was measured on carboxymethyl cellulose (CMC), Whatman filter paper, cotton, Avicel PH-101, respectively. All reactions were done in 50 mM acetic acid–sodium acetate buffer (pH 5.0) at 55 ℃ for 30 min, and the total reaction volume was 200 μL. The final concentration of Pp-ArCel5, Pp-ArCel5-NG, Pp-ArCel5-LD, Pp-ArCel5-D used in the assay was 0.1 mg/mL, and substrate concentration was 10 mg/mL. The release of reducing sugar was measured by the 3,5-dinitrosalicylic acid (DNS) method with glucose as a standard (Miller 1959). One unit of enzyme activity was defined as the amount of enzyme that produced 1 μmol reducing sugar per minute.

### Morphology analysis

The system contained a piece of filter paper (1 cm × 2 cm) and 2.5 mg protein in 5 mL acetic acid–sodium acetate buffer (50 mM, pH 5.0), and the mixture was statically incubated at 55 ℃ for 24 h. After being treated with Pp-ArCel5, Pp-ArCel5-NG, Pp-ArCel5-LD, Pp-ArCel5-D and BSA, respectively, the morphology of the filter papers was observed.

### Hydrature index analysis

The system contained 200 mg filter paper and 2.5 mg protein in 5 mL acetic acid–sodium acetate buffer (50 mM, pH 5.0), and the mixture was statically incubated at 55 ℃ for 24 h. After being treated with Pp-ArCel5, Pp-ArCel5-NG, Pp-ArCel5-LD, Pp-ArCel5-D and BSA, respectively, removed the enzyme solution on the surface of the filter paper, the filter paper was weighed as wet weight (*W*_w_). Its dry weight (*W*_d_) was obtained when the treated filter paper was dried at 60 ℃ to the constant weight after being washed three times with distilled water. The dried filter paper after treated with Pp-ArCel5, Pp-ArCel5-NG, Pp-ArCel5-LD, Pp-ArCel5-D and BSA was abbreviated as FP_WT_, FP_NG_, FP_LD_, FP_CD_ and FP_BSA_, respectively. The hydrature index (HyI) (Lan et al. 2016) was calculated as (Eq. 1):

1 $${\text{HyI}}\% = \frac{{W_{{\text{w}}} - W_{{\text{d}}} }}{{W_{{\text{d}}} }} \times 100.$$

The dried filter paper was subjected to SEM, XRD, FTIR, and synergistic effect analysis separately.

### XRD analysis

The crystalline index of FP_WT_, FP_NG_, FP_LD_, FP_CD_ and FP_BSA_ were analyzed by X-ray diffraction (XRD) using a Rigaku D/Max-3B diffractometer (Tokyo, Japan) with Cu Kα radiation (*λ* = 0.154 nm). 30 kV accelerating voltage and 30 mA current. The samples were detected in the range of 2*θ* between 5° and 50° with a scanning velocity of 2° min^−1^. The crystalline index (CrI) (Park et al. 2010) was calculated according to the following equation (Eq. 2):

2 $${\text{CrI }}(\% ) = (1 - {\text{ham}} / {\text{hcr}}) \times 100,$$

in which hcr is the peak height at 2*θ* = 22.5° and ham is the peak height at 2*θ* = 18°.

### FTIR analysis

The Fourier transformed infrared spectroscopy (FTIR) spectral ranges were recorded from 400 to 4000 cm^−1^ using Spectrum One-B spectrometer (PerkinElmer, USA). 1 mg of FP_WT_, FP_NG_, FP_LD_, FP_CD_ and FP_BSA_ mixing with 100 mg of spectroscopic grade KBr, pressed into pellets and then placed into the plate that was cleaned with acetone twice. Spectrometer with the detector at 4 cm^−1^ and 100 scans per sample were signal-averaged and stored.

### Synergistic action between Pp-ArCel5 mutants and cellulase

All reactions were done in 50 mM acetic acid–sodium acetate buffer, pH 5.0, at 55 °C with shaking 120 rpm over 24 h, and the total reaction volume was 200 μL. The final concentration of FP_WT_, FP_NG_, FP_LD_, FP_CD_, FP_BSA_ and FP (untreated filter paper) used in the assay was 10 mg/mL, and *Aspergillus niger* cellulase (Sigma-Aldrich, St. Louis, USA) concentration was 20 µg/mg substrate. The FP_BSA_ and FP treated by fungal cellulase were used as the control experiment.

### Statistical analysis

All analyses were performed in triplicate, and all data were expressed as the means ± standard errors of the means. Data were subjected to one-way analysis of variance (ANOVA). A *P*-value < 0.05 was considered to be significant, following SPSS 11 (SPSS Inc., Chicago, IL, USA) software analysis.

## Results and discussion

### Isolation of a potential accessory protein

**Fig. 1:**
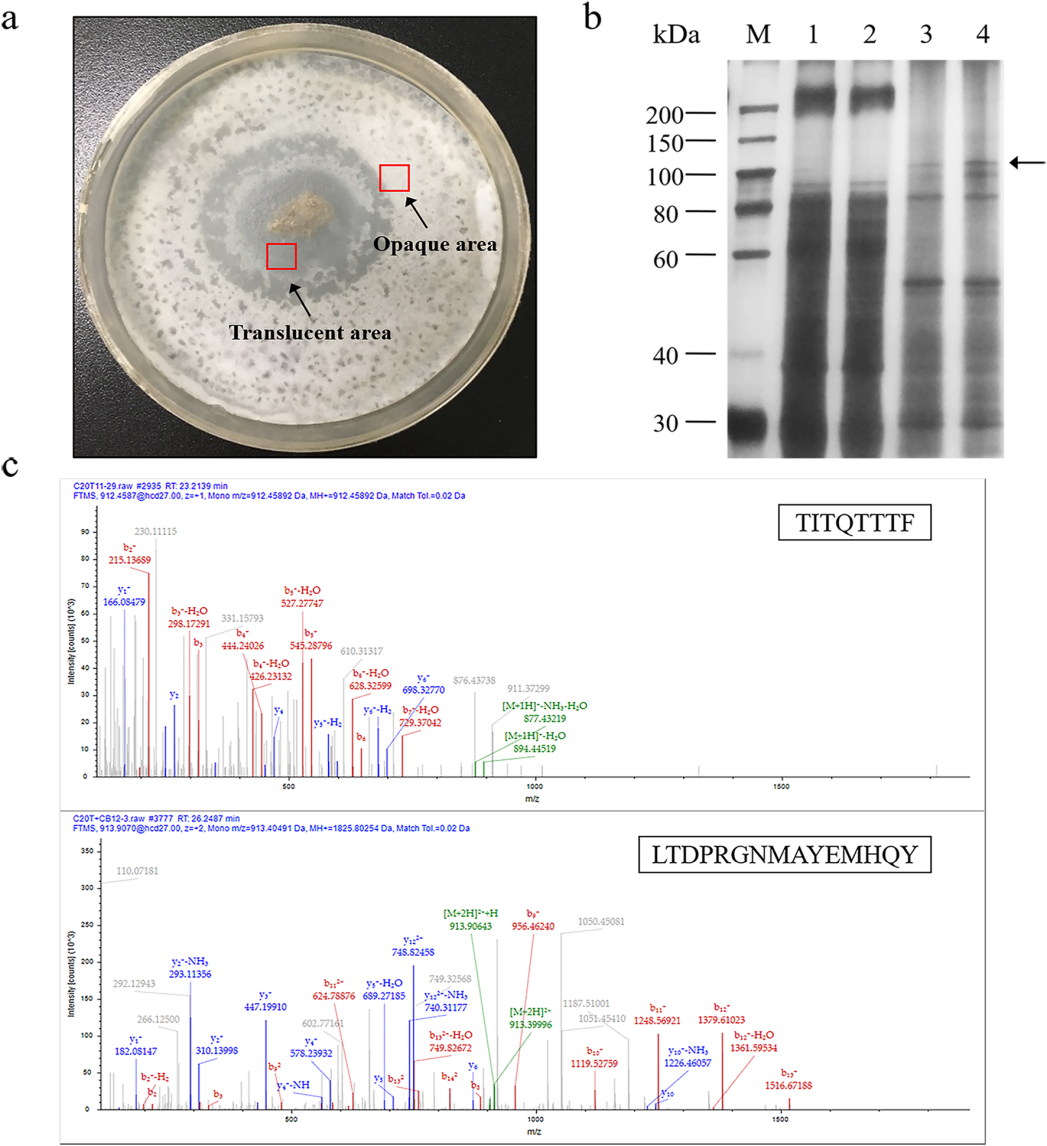
Isolation of ArCel5. **a** The decrystallization phenomenon on filter paper agar plate caused by the growth of cellulose-gelatinizing fungus *Arthrobotrys* sp.CX1. **b** The different secretary proteins between opaque area and the transparent area were assessed by SDS-PAGE. Lanes 1–2 showed the total proteins of the opaque area in the filter paper sample. Lanes 3–4 showed the total proteins of the transparent area in the filter paper sample. The arrow indicates a differential protein band found only in the transparent region. **c** LC–MS/MS fragment ion spectra of the peptides in the arrow area. The peptide TITQTTTF and LTDPRGNMAYEMHQY were distinguished and identified

Previously, a cellulose-gelatinizing fungus *Arthrobotrys* sp. CX1 was isolated from a soil sample. *Arthrobotrys* sp. CX1 could make the filter paper translucent by gelatinization, swell the compact structure in filter paper cellulose, reduce the water-assisted decrystallization of the crystalline structure by breaking hydrogen bonds, and improve filter paper cellulose degradability. It was speculated that strain CX1 could secrete some accessory proteins in the translucent filter paper that had strong activity on cellulose decrystallization (Lan et al. 2016). To isolate the key accessory protein, *Arthrobotrys* sp. CX1 was cultured on the filter paper agar plate, secretary proteins in the transparent and opaque areas of filter paper were analyzed using SDS-PAGE. As shown in Fig. 1a, one differential protein of about 100 kDa was detected in the transparent area of the filter paper sample. After being digested with trypsin, the tryptic peptides of the differential protein were analyzed by LC–MS/MS fragment ion spectra (Fig. 1b). Two sequences TITQTTTF and LTDPRGNMAYEMHQY were preliminarily identified, showing precursor ions at MH^+^ 912.5 and MH^+^ 1825.8 in the MS spectrum (Fig. 1c). Gene of the differential protein contains 1269 bp, with a GC content of 52.96%. The coding gene of the differential protein was annotated as a hypothetical protein (GenBank: MN654111).

### Sequence analysis and homology-modeling of ArCel5

**Fig. 2:**
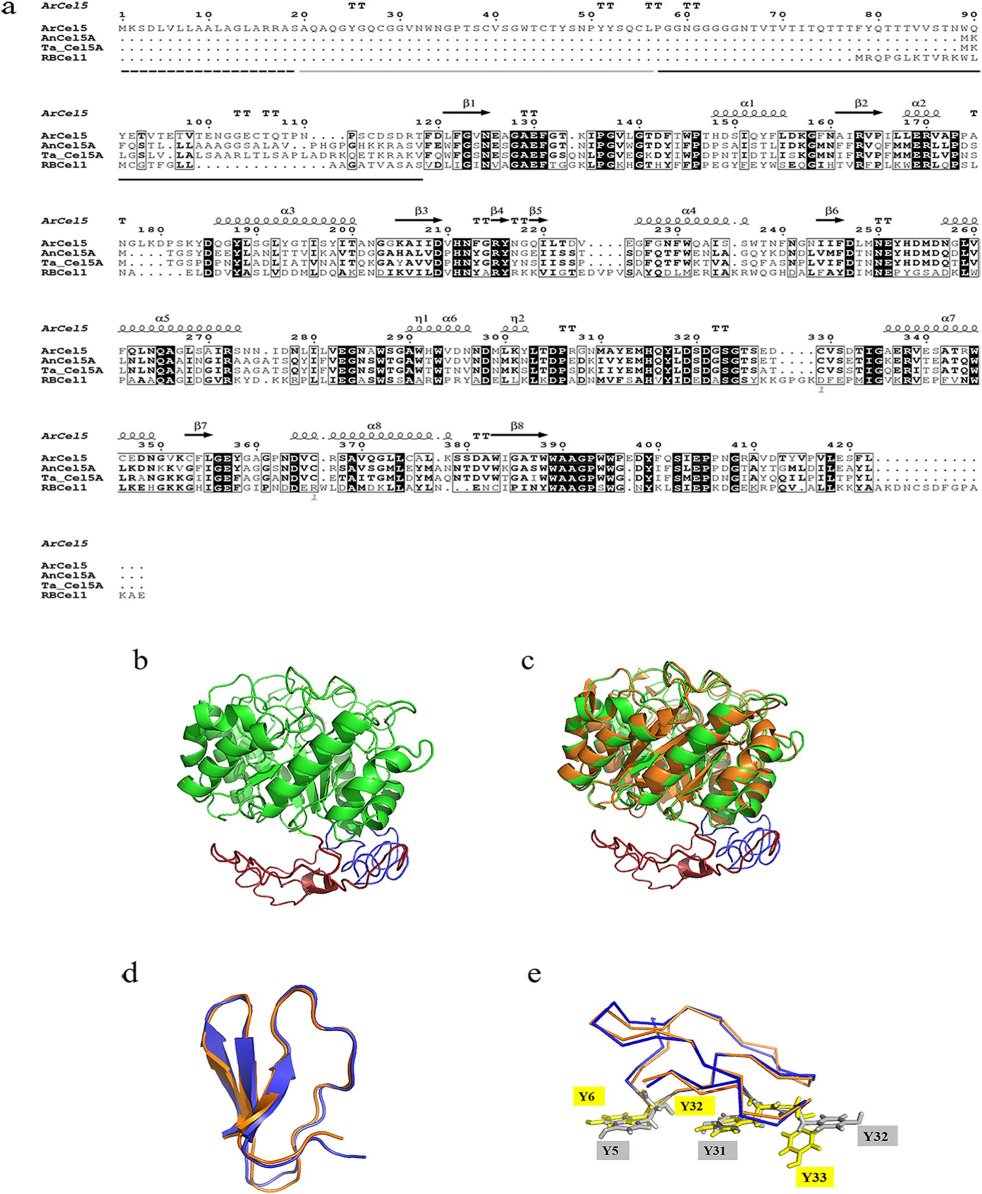
Sequence and structure properties of ArCel5. **a** Alignment of amino acid sequences of ArCel5 (GenBank accession number MN654111), Ta_Cel5A (GenBank accession number AAL88714.2), AnCel5A (GenBank accession number AF331518.1) and RBCel1(GenBank accession number ACO55737). The signal peptide (dotted line), family 1 carbohydrate-binding molecule (gray full line) and linker peptide (black full line) of ArCel5 were also marked. The amino acid residues conserved in all four proteins are shown with white letters and black boxes, and the amino acid residues conserved in two or three differential protein are shown with highlighted in gray, and the secondary structure is shown above the sequence. Sequence alignment was analyzed by ClustalOmega (Petersen et al. 2011), and results were output by Espript 3 server (Patrice et al. 2003). **b** ArCel5 structure was shown in a cartoon model with the CBM, linker, and CD domain in blue, red and green, respectively. **c** A structural overlay of the modeled ArCel5 with AnCel5A (PDB entry 5i77, orange) was made to allow visual comparison. **d** Structure comparison of TrCBM1 (PDB entry 1CBH, orange) with ArCel5-CBM (blue). **e** ArCel5-CBM and TrCBM1 could form a flat binding surface with cellulose, and the three conserved tyrosines were shown in yellow and gray, respectively. The structure homology-modeling of ArCel5 was predicted by using I-TASSER online server (http://zhanglab.ccmb.med.umich.edu/I-TASSER/)

To further speculate the function of the isolated hypothetical protein, sequence analysis and homology-modeling were performed. As shown in Fig. 2a, the signal peptide of the hypothetical protein was composed of 19 amino acid residues predicted by Signal P (Version 4.1). The mature protein contained 403 amino acids with a predicted molecular mass of about 44 kDa. The hypothetical protein showed the highest identity scores with three characterized glycoside hydrolase family 5 (GH5) enzymes, including 1,4-β-endoglucanase AnCel5A from *Aspergillus niger* (accession number AF331518.1, 49% identity), endoglucanase Ta_Cel5A from *Thermoascus aurantiacus* (accession number AAL88714.2, 48% identity), and endoglucanase RBCel1 from a soil metagenome library in the Antarctic (accession number ACO55737, 33% identity) (Delsaute et al. 2013; Leggio and Larsen 2002; Yan et al. 2016). The secondary structure prediction of the hypothetical protein revealed a multi-domain structure, consisting of an N-terminal CBM domain (Ala^20^ to Leu^56^), a linker region containing 63 amino acids, and a catalytic domain (Phe^119^ to Leu^479^) at the C terminus. The catalytic domain of the hypothetical protein contained a (β/α)8-TIM barrel structure, which was composed of an eight-stranded parallel β-sheet surrounded by eight α-helices (Fig. 2a), indicating a typical structure of the catalytic domains of GH5 family (Payne et al. 2015). Also, E232 and E338 of the central barrel were identified as catalytic residues in the hypothetical protein based on previous analysis (Payne et al. 2015). Therefore, the identified hypothetical protein was designated as ArCel5.

Homology modeling was performed to predict the typical GH5 structure of ArCel5. The structural superposition of the modeled ArCel5 with AnCel5A (PDB ID: 5i77) revealed that the catalytic domain of ArCel5 is nearly identical to that of 5i77 with a typical GH5 structure (Fig. 2b, c). Sequence analysis showed that ArCel5 has an extra CBM-linker region than all three typical GH5 enzymes. The 37 amino acids at the N-terminal of ArCel5 was classified as CBM1 family mainly derived from fungal cellulase (Guo and Catchmark 2013; Linder et al. 1995), such as the cellobiohydrolase from *Trichoderma reesei*, which was investigated to bind cellulose substrate through *Tr*CBM1 (PDB ID: 1CBH) (Kraulis et al. 1989; Tomme et al. 1998). Sequence alignment showed that ArCel5-CBM share 69% identity with *Tr*CBM1, which contains three conserved tyrosines (Fig. 2d, e) (Linder et al. 1995). Currently there is no report about accessory proteins that belong to the GH5 family. Therefore, it is speculated that different from other GH5 proteins, ArCel5 might possess novel properties.

### ArCel5 is glycosylated at the linker region

Previous researches revealed that glycosylation modification plays an essential role in the function of glycosidases, especially in enhancing enzyme stability and the binding affinity between proteins and substrates (Amore et al. 2017; Badino et al. 2017; Payne et al. 2013). Here, the glycosylation sites of ArCel5 were predicted by NetOGlyc (http://www.cbs.dtu.dk/services/NetOGlyc/) and NetNGlyc (http://www.cbs.dtu.dk/services/NetNGlyc/) servers. Totally 16 threonine sites in the linker of ArCel5 were identified as candidate O-glycosylation sites, and no *N*-/*O*-glycosylation sites were found in the CBM1 and GH5 domains. Differently, highly homologous proteins AnCel5A, Ta_Cel5A, RBCel1 are *O*-glycosylated and *N*-glycosylated in the GH5 domain, suggesting that ArCel5 might function in different ways.

**Fig. 3:**
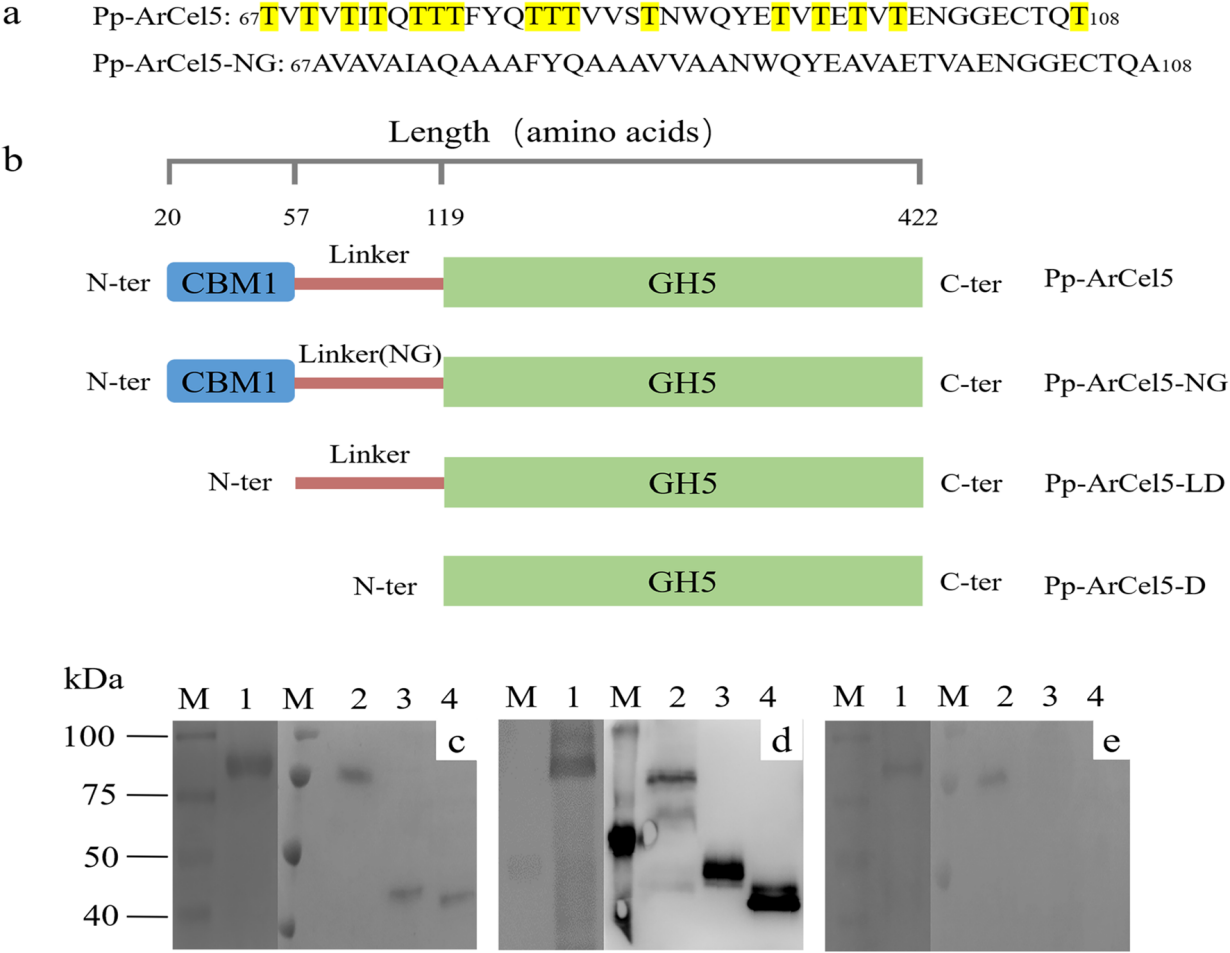
Rational design and SDS-PAGE analysis of ArCel5 mutants. **a** Sequences of the linker regions from the 67th base to the 108th base. **b** Constructions of the mutants with variable lengths. **c** Coomassie stain, **d** western blot analysis, **e** glycoprotein stain. Lanes 1: Pp-ArCel5; lanes 2: Pp-ArCel5-LD; lanes 3: Pp-ArCel5-NG; lanes 4: Pp-ArCel5-D

To further confirm the glycosylation modification existed in ArCel5, two recombinants Pp-ArCel5 and Ec-ArCel5 were heterologously expressed in *P. pastoris* X-33 and *E. coli* BL21(DE3), respectively. Compared with Ec-ArCel5, wild-type ArCel5 and Pp-ArCel5 showed higher molecular weight (Fig. 1; Additional file 1: Fig. S1), which might be attributed to different post-translational modification (PTM) levels of proteins among different strains (Amore et al. 2017; Beckham et al. 2012). To verify the location of glycosylation sites, three recombinants including Pp-ArCel5-NG (all the Thr residues in the linker were mutated to Ala), Pp-ArCel5-LD (mutant without CBM1) and Pp-ArCel5-D (mutant without both CBM1 and linker) were constructed (Fig. 3a, b). Different from Pp-ArCel5-NG and Pp-ArCel5-D, Pp-ArCel5 and Pp-ArCel5-LD migrated as diffused bands with an apparent molecular weight of higher than 75 kDa, which was higher than their theoretical molecular weight (Fig. 3c, d). As shown in the glycosylation stained PAGE, both Pp-ArCel5-D and Pp-ArCel5-NG exhibited no stained band, whereas a clear glycoprotein band of Pp-ArCel5 and Pp-ArCel5-LD could be observed (Fig. 3e). All these results demonstrated that Pp-ArCel5 was strongly glycosylated, and the glycosylation sites were only located in the linker region, which was consistent with the glycosylation prediction result.

### CBM1 and glycosylation of linker promote the absorption capacity of Pp-ArCel5

The adsorption of proteins to cellulose is an essential step for effective pretreatment of cellulose (Johns et al. 2017; Nidetzky et al. 1994). To determine the influence of different domains and glycosylation on the affinity of ArCel5, the adsorption capacity of different recombinants towards cellulosic substrates was analyzed. The fitted Langmuir isotherm results are shown in Additional file 1: Fig. S2 and the corresponding parameters are summarized in Table 1. *B*_max_/*K*_d_ was applied to quantify the affinity of the protein to cellulose (Arola and Linder 2016; Christensen et al. 2020). The *B*_max_/*K*_d_ of Pp-ArCel5 and Pp-ArCel5-NG towards filter paper (CrI ≈ 84%) was significantly higher than that towards Avicel (CrI ≈ 77%), due to difference crystallinity of substrates (Hall et al. 2010). Comparatively, no significant difference was observed in *B*_max_/*K*_d_ values when Pp-ArCel5-LD and Pp-ArCel5-D were incubated with Avicel PH-101 and filter paper, respectively. These results revealed that CBM1 might belong to type A CBMs that could interact with the multiple planar cellulose chains found in crystalline cellulosic substrates (Hashimoto 2006; Mccartney et al. 2004; McLean et al. 2002), and helped Pp-ArCel5 bind to filter paper with higher crystallinity. The *B*_max_/*K*_d_ values of Pp-ArCel5, Pp-ArCel5-NG, Pp-ArCel5-LD and Pp-ArCel5-D toward both filter paper and Avicel were decreased in turn, suggesting that CBM and the linker could promote the binding affinity of Pp-ArCel5 to cellulosic substrates, and glycosylation of the linker played a positive role in the binding process. Previous researches revealed that CBM bind to cellulose with a hydrophobic surface (Nakamura et al. 2016, Linder et al. 1995; Guo and Catchmark 2013), and glycosylation enhance the binding ability of the linker to water, which subsequently causes the linker to bind to the hydrophilic surface of the cellulose crystalline region (Nakamura et al. 2016). Our conclusion was similar to previous studies, which also revealed that CBMs and linker glycosylation facilitate the binding of enzymes toward substrates (Badino et al. 2017; Gilbert et al. 2013; Payne et al. 2015, 2013).

**Table 1:** Adsorption parameters of ArCel5 constructions on Avicel pH-101 and filter paper

Substrate	Avicel pH-101					Filter paper				
	ArCel5	ArCel5-LD		ArCel5-NG	ArCel5-D	ArCel5	ArCel5-LD		ArCel5-NG	ArCel5-D
*B*_max_(μmol/g)	0.25 ± 0.03	0.18 ± 0.01		0.33 ± 0.10	0.12 ± 0.00	0.34 ± 0.03	0.15 ± 0.00		0.46 ± 0.022	0.21 ± 0.00
*K*_d_ (μM)	0.48 ± 0.11	2.08 ± 0.30		1.41 ± 0.19	2.53 ± 0.24	0.27 ± 0.05	1.79 ± 0.19		1.08 ± 0.11	2.04 ± 0.19
Absolute specificity (L/g)^a^	0.52	0.09		0.23	0.05	1.26	0.083		0.43	0.10
Relative specifity^b^	10.4	1.8		4.6	1	12.6	0.83		4.3	1

*B*_max_ maximum binding capacity, *K*_d_ dissociation constant

^a^*B*_max_/*K*_d_

^b^Absolute specificities normalized on ArCel5-D

### CBM1 and glycosylation of Pp-ArCel5 linker dramatically affect the structure of filter paper

**Fig. 4:**
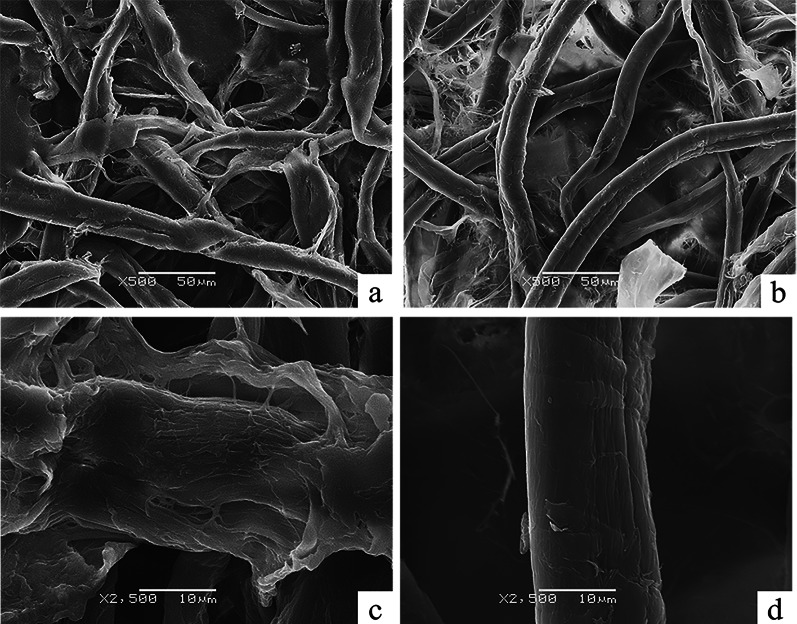
Scanning electron microscopy of filter paper after being treated by different solutions. Pictures with two different scale markers were taken at × 500 and × 2500 magnifications. **a**, **c** Filter paper treated by BSA in 50 mM acetic acid–sodium acetate buffer (pH 5.0) with different magnification. **b**, **d** Filter paper treated by Pp-ArCel5 in 50 mM acetic acid–sodium acetate buffer (pH 5.0) with different magnification. The final concentration of filter paper with 40 g/L was incubated in 50 mM acetic acid–sodium acetate buffer (pH 5.0), containing 0.5 g/L Pp-ArCel5 or BSA at 55 ℃ for 24 h

To preliminarily verify the function of Pp-ArCel5, the microstructure features of filter paper treated by Pp-ArCel5 were observed using SEM. As shown in Fig. 4, after being incubated with Pp-ArCel5, microfibrils in filter paper became dissociated and expanded, creating an amorphic surface and wider width (Fig. 4a, c). The observation was similar to the microstructure of translucent filter paper treated by the strain CX1 (Lan et al. 2016). Differently, after pretreatment with BSA the microfibrils with a smooth surface were not dispersed (Fig. 4b, d). These results indicated that Pp-ArCel5 could disrupt the surface structure of cellulosic substrates. A similar phenomenon was observed previously (Jäger and Fischer 2011; Hall et al. 2011). As shown in Additional file 1: Table S2, Pp-ArCel5 has lower specific hydrolysis activity on filter paper, cotton, and Avicel than CMC. Some swollenins and expansins also have lower specific hydrolysis activity and the ability to destroy the structure of cellulose (Sdrobi et al. 2011, Jäger and Fischer 2011; Hall et al. 2011). Based on these results, ArCel5 might function as a novel accessory protein.

**Fig. 5:**
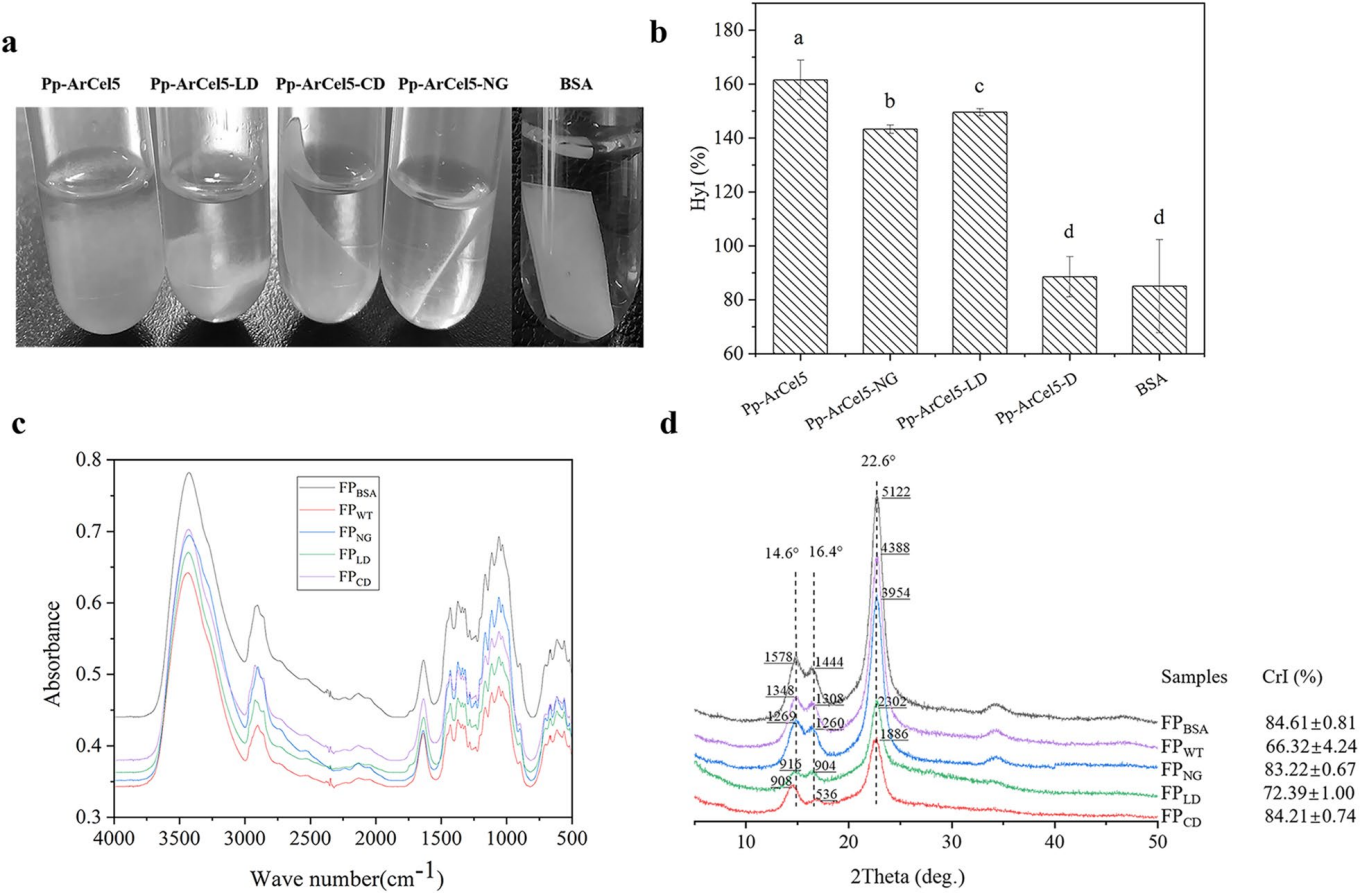
Summarized properties of filter paper after being treated by different solutions. **a** Filter paper was deconstructed by ArCel5 mutants. Pp-ArCel5, Pp-ArCel5-NG, Pp-ArCel5-D or BSA acted on filter paper for 48 h to observe disruption. The filter paper with a size of 1 cm × 2 cm was incubated in 50 mM acetic acid–sodium acetate buffer (pH 5.0), containing 0.5 g/L ArCel5 mutants or BSA at 55 ℃ for 24 h. **b** The hydrature index of filter paper after being treated by ArCel5 mutants and BSA. The different letters mean significant difference, during the same letter means no significant difference. The final concentration of filter paper with 40 g/L was incubated in 50 mM acetic acid–sodium acetate buffer (pH 5.0) containing 0.5 g/L ArCel5 mutants or BSA at 55 ℃ for 24 h. **c** FTIR spectroscopy spectrum of the filter paper after being treated by the ArCel5 mutants or BSA. **d** The X-ray diffraction spectrum imbibed of the filter paper after being treated by ArCel5 mutants and BSA

After being treated by Pp-ArCel5 mutants, the morphology of the filter papers was investigated. As shown in Fig. 5a, the Pp-ArCel5 caused the maximum degree of deagglomeration of the filter paper. Comparatively, Pp-ArCel5-LD and Pp-ArCel5-NG showed weaker swelling activity against the filter paper. There was no significant structural change of filter paper treated by Pp-ArCel5-D or BSA. The result indicated that the deagglomeration ability of ArCel5 against filter paper could be weakened by removing CBM1 or glycosylated linker. The hydrature index (HyI) values of FP_WT_, FP_NG_, FP_LD_, respectively, increased by 89.4%, 68.2%, 76.5% in comparison with that of FP_BSA_, whereas the HyI of FP_CD_ has no significant difference when compared with that of the control (Fig. 5b). The increase of HyI might be caused by water penetration into the microfibrils and cellulose amorphogenesis (Lan et al. 2016), which is similar to the swelling degree (Qs) indicating the swelling characteristics of cellulose (Sdrobi et al. 2011). The result suggested that both CBM1 and glycosylated linker had a significant impact on increasing the water absorption capacity of filter paper by Pp-ArCel5. HyI results of filter paper treated by different Pp-ArCel5 mutants were consistent with the morphology investigation, revealing that the water absorption capacity of filter paper caused by Pp-ArCel5 directly related with the deagglomeration degree of filter paper, the similar finding has been reported previously (Lan et al. 2016; Sdrobi et al. 2011; Tsuchida et al. 2014). Moreover, both CBM1 and O-glycosylated linker were essential for the function of Pp-ArCel5.

To further determine the relationship between the water absorption and structure of filter paper after being treated by different Pp-ArCel5 mutants, the FT-IR and XRD were performed. It is known that the increase of hydrogen bonds could enhance the crystallinity and mechanical strength of cellulose (Poletto et al. 2012; Popescu et al. 2011). As shown in Fig. 5c, the absorbance values of the hydrogen-bonded OH stretching of cellulose varied from 4000 to 2995 cm^−1^ (Haque et al. 2015), and the characteristic peaks of cellulose crystallization were located at 2900 cm^−1^, 1430 cm^−1^, 1375 cm^−1^ regions (Sdrobi et al. 2011). In the FT-IR spectrum, the strongest absorption peaks at 3430 cm^−1^, 2900 cm^−1^, 1430 cm^−1^ and 1375 cm^−1^ of FP_WT_, FP_NG_, FP_LD_, FP_CD_ were lower than that of the FP_BSA_, which indicated that after being treated by Pp-ArCel5 mutants, the hydrogen bonds of filter paper were reduced, and the structure of filter paper became loose and disordered (Lan et al. 2016; Qin et al. 2013). As shown in Fig. 5d, characteristic peaks at 14.6°, 16.4°, and 22.6° of diffraction angles 2*θ* corresponded to 010, 10ī, and 002 crystal planes of cellulose I (Eibinger et al. 2016; Hall et al. 2011). After being treated by ArCel5 mutants, no obvious change in peak shape and position of the filter papers could be observed, indicating that the crystal form of the filter papers remained type I structure (Park et al. 2010) (Cheng et al. 2011). Compared with the CrI of FP_WT_, the CrI value of FP_LD_, FP_NG_, FP_CD_ increased in ture when CBM1, glycans and linker were removed from Pp-ArCel5. These findings were also consistent with the observations in HyI values and morphology of filter paper, suggesting that Pp-ArCel5 could effectively destroy hydrogen bonds and decrease the crystallinity of filter paper by increasing the water absorption capacity, which further led to significant deagglomeration of filter paper. During this process, both CBM and glycosylated linker in Pp-ArCel5 played important roles. Similar processes have been investigated previously (Jäger et al. 2011; Qin et al. 2013).

### Glycosylation of linker promotes the synergism between Pp-ArCel5 and cellulase

**Fig. 6:**
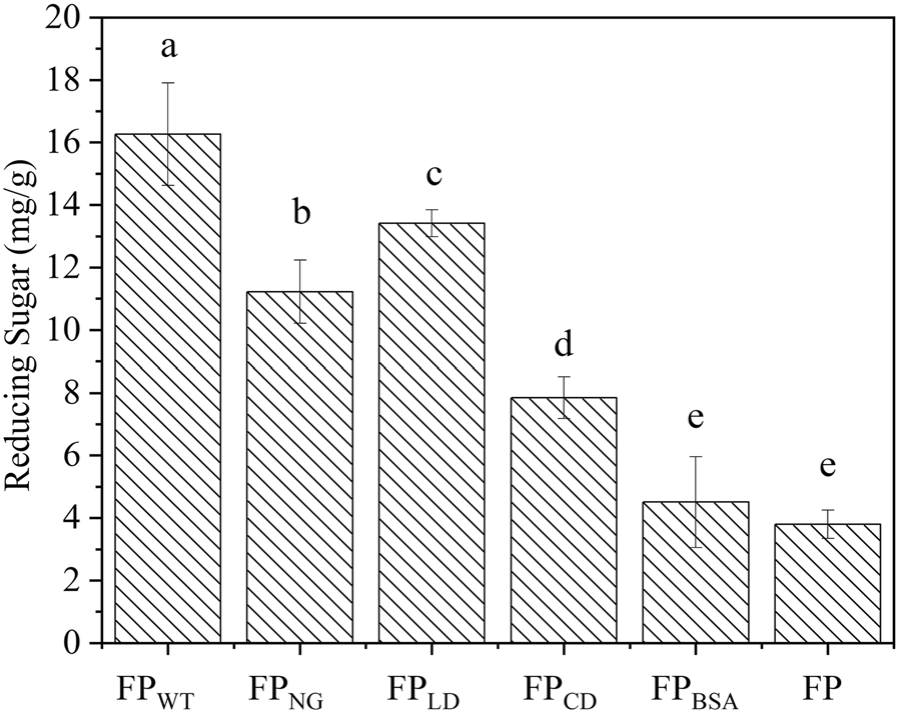
The synergy between ArCel5 mutants and fungal cellulase in the hydrolysis of filter paper. All reactions were done in 50 mM acetic acid–sodium acetate buffer (pH 5.0) at 55 ℃ with shaking 120 rpm over 24 h. The final concentration of filter paper treated in different ways was all 10 mg/mL, and *Trichoderma reesei* cellulase was 20 µg/mg substrate. The FP_BSA_ treated by fungal cellulase was used as reference experiment. Error bars show the standard deviation of three independent experiments

Previous reports showed that the enzymolysis efficiency of cellulose could be affected by the physical structure of the substrate (Kumar and Wyman 2009; Zhang and Lynd 2004). Therefore, the role of CBM and glycosylated linker of Pp-ArCel5 in synergistic action with cellulase during the hydrolysis process of filter paper was investigated. As shown in Fig. 6, compared to the single cellulase hydrolysis system, no significant difference was observed in reducing sugars values after filter paper being treated by BSA. However, more reducing sugars were released from filter paper after being incubated with Pp-ArCel5 recombinants. Furthermore, the content of released reducing sugars were reduced in turn when cellulase was cooperated with Pp-ArCel5, Pp-ArCel5-LD, Pp-ArCel5-NG and Pp-ArCel5-D, respectively. The result demonstrated that Pp-ArCel5 could effectively help cellulase to degrade filter paper, and both CBM and linker in Pp-ArCel5 were essential for this synergistic process. Moreover, the function of linker region was strengthened by glycosylation modification. This investigation could be attributed to our previous speculation that CBM1 and glycosylation of linker dramatically affects the structure of filter paper. According to our knowledge, this is the first report that glycosylation modified linker region could promote the synergistic effect of accessory protein on cellulose hydrolysis.

## Conclusions

ArCel5 is an accessory protein obtained from a cellulose-gelatinizing fungus *Arthrobotrys* sp. CX1, and probably belongs to a novel protein of the GH5 family. ArCel5 could efficiently disrupt the structure of cellulose and have lower specific hydrolysis activity on cellulosic substrates. The CBM1 and linker played key roles in filter paper decrystallization, which could be further strengthened by the glycosylation of the linker. Overall, these results highlight the importance of the multi-domain structure and post-translational modification in accessory protein.

### Supplementary Information

**Additional file 1: Table S1** Sequences of primers used in recombinant vector construction. **Table S2 **The specific activity of ArCel5 mutants. **Fig. S1** SDS-PAGE analysis of Ec-ArCel5 and Pp-ArCel5. M, molecular mass marker (in kilodaltons); lanes 1, Pp-ArCel5; lanes 2, Ec-ArCel5. **Fig. S2** Adsorption isotherms of purified proteins on filter paper and Avicel. Experiments were done at 25℃ in 50 mM acetic acid-sodium acetate buffer, pH 5.0, over 1 h with 120 rpm. Substrate concentration was 10 mg/mL in a total reaction volume of 500 µL. The corresponding parameters, maximum binding capacity related to the unit mass of substrate (*B*_max_) and the dissociation constant *K*_d_, are summarized in Table 1

## Data Availability

The dataset (graphs and tables) supporting the conclusions of this article is available.
