# Supplementary material for: A novel accessory protein ArCel5 from cellulose-gelatinizing fungus Arthrobotrys sp. CX1
Source: Bioresour Bioprocess. 2022 Mar 21;9(1):27. doi: 10.1186/s40643-022-00519-1 (PMC10991334; doi:10.1186/s40643-022-00519-1)
Supplement: Supplementary file 1 — Additional file 1: Table S1 Sequences of primers used in recombinant vector construction. Table S2 The specific activity of ArCel5 mutants. Fig. S1 SDS-PAGE analysis of Ec-ArCel5 and Pp-ArCel5. M, molecular mass marker (in kilodaltons); lanes 1, Pp-ArCel5; lanes 2, Ec-ArCel5. Fig. S2 Adsorption isotherms of purified proteins on filter paper and Avicel. Experiments were done at 25℃ in 50 mM acetic acid-sodium acetate buffer, pH 5.0, over 1 h with 120 rpm. Substrate concentration was 10 mg/mL in a total reaction volume of 500 µL. The corresponding parameters, maximum binding capacity related to the unit mass of substrate (Bmax) and the dissociation constant Kd, are summarized in Table 1 [file 40643_2022_519_MOESM1_ESM.docx]

**Supplementary material**

**A novel accessory protein ArCel5 from cellulose-gelatinizing fungus *Arthrobotrys* sp. CX1**

Yue Yuan ^#^, Chunshu Chen ^#^, Xueyan Wang, Shaonian Shen, Xiaoyu Guo, Xiaoyi Chen, Fan Yang *, Xianzhen Li *

School of Biological Engineering, Dalian Polytechnic University, Ganjingziqu, Dalian 116034, People’s Republic of China

**Table S1** Sequences of primers used in recombinant vector construction

| **Name** | **Sequence** | **Description** |
| --- | --- | --- |
| ArCel5 fw | 5'-CGGAATTCGCCCAAGAGTCTCGGGGTTACGGAC-3' | Construction primer of expression vector pPICZαA-ArCel5 (*Eco*R I restriction site underlined) |
| ArCel5 rev | 5'-GCTCTAGACGCAAGAAAGACTCAAGAACTGG-3' | Construction primer of expression vector pPICZαA-ArCel5 (*Xba* I restriction site underlined) |
| ArCel5-LD fw | 5'-GGAATTCCCAGGAGGTAATGGTGGTGGTGG-3' | Used as primers to construct the expression vector pPICZαA-ArCel5-LD with ArCel5 rev (*Eco*R I restriction site underlined) |
| ArCel5-D fw | 5'-GGAATTCGACCTCTTCGGTGTCAACGAGGC-3' | Used as primers to construct the expression vector pPICZαA-ArCel5-D with ArCel5 rev (*Eco*R I restriction site underlined) |
| ArCel5 rev1 | 5'-GCAAGCTTTTACAAGAAAGACTCAAGAACTGG-3' | Used as primers to construct the expression vector pET28a-ArCel5 with ArCel5 rev (*Hin*d III restriction site underlined) |
| NG fw | 5'-CATACTCCAACCCTTACTA-3'， | Construction primer of expression vector pPICZαA-ArCel5-NG |
| NG rev | 5'-GCACCGGCCTCGTTGAC-3' | Construction primer of expression vector pPICZαA-ArCel5-NG |

**Table S2** The specific activity of ArCel5 mutants

| **Mutants** | **CMCase (U/mg)** | **Filter paper (U/mg)** | **Cotton (U/mg)** | **Avicel (U/mg)** |
| --- | --- | --- | --- | --- |
| Pp-ArCel5 | 2.00±0.08 | 0.13±0.01 | 0.12±0.00 | 0.07±0.01 |
| Pp-ArCel5-NG | 0.63±0.04 | 0.11±0.01 | 0.07±0.01 | 0.02±0.00 |
| Pp-ArCel5-LD | 0.76±0.07 | 0.02±0.00 | 0.02±0.00 | 0.05±0.00 |
| Pp-ArCel5-D | 0.49±0.03 | 0.09±0.01 | 0.07±0.00 | 0.01±0.00 |


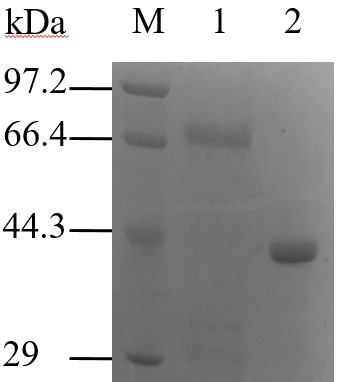


Fig. S1.

**Fig. S1** SDS-PAGE analysis of Ec-ArCel5 and Pp-ArCel5. M, molecular mass marker (in kilodaltons); lanes 1, Pp-ArCel5; lanes 2, Ec-ArCel5.


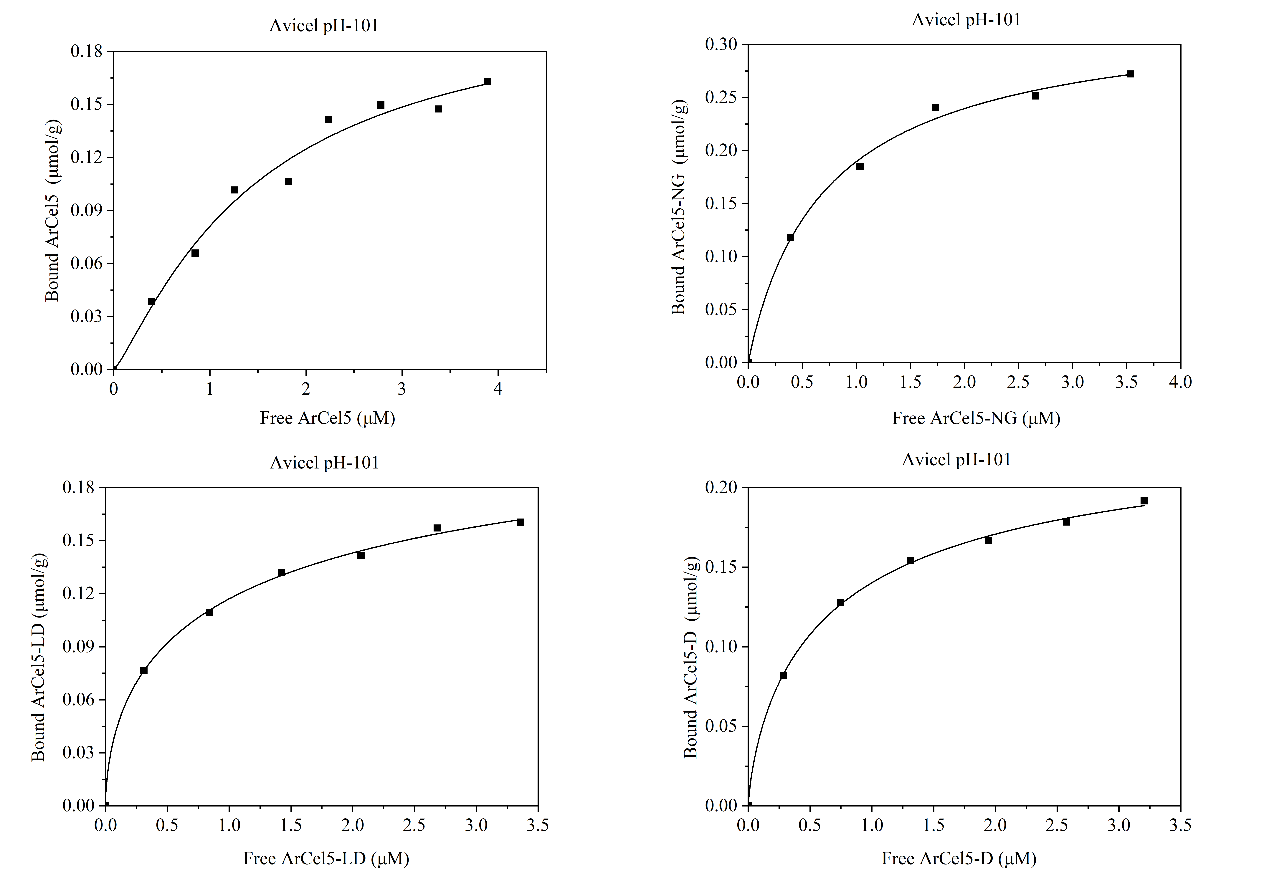


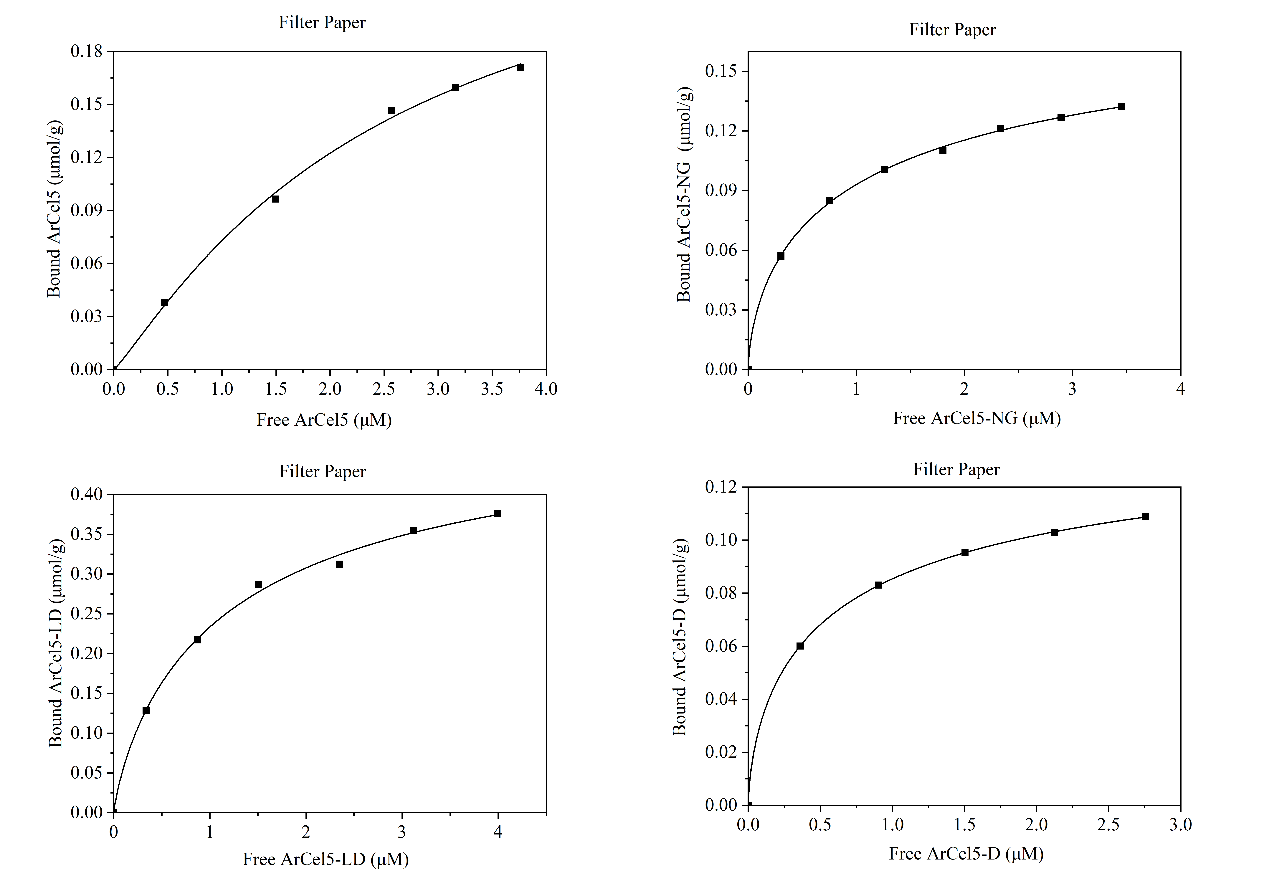


**Fig. S2** Adsorption isotherms of purified proteins on filter paper and Avicel. Experiments were done at 25℃ in 50 mM acetic acid-sodium acetate buffer, pH 5.0, over 1 h with 120 rpm. Substrate concentration was 10 mg/mL in a total reaction volume of 500 µL. The corresponding parameters, maximum binding capacity related to the unit mass of substrate (*B*_max_) and the dissociation constant *K*_d_, are summarized in Table 1.
